# Improving Release of Liposome-Encapsulated Drugs with Focused Ultrasound and Vaporizable Droplet-Liposome Nanoclusters

**DOI:** 10.3390/pharmaceutics13050609

**Published:** 2021-04-22

**Authors:** Arvin Honari, Darrah A. Merillat, Aditi Bellary, Mohammadaref Ghaderi, Shashank R. Sirsi

**Affiliations:** 1 Department of Bioengineering, Erik Johnson School of Engineering, The University of Texas at Dallas, Richardson, TX 75080, USA; Arvin.Honari@utdallas.edu (A.H.); Darrah.Merillat@utdallas.edu (D.A.M.); Aditi.Bellary@utdallas.edu (A.B.); Mohammadaref.Ghaderi@UTDallas.edu (M.G.); 2 Department of Radiology, The University of Texas Southwestern Medical Center, 5323 Harry Hines Blvd, Dallas, TX 75390, USA

**Keywords:** acoustic droplet vaporization, acoustic targeting, ultrasound-mediated drug delivery, focused ultrasound, drug uncaging, phase-shift contrast agents

## Abstract

Active targeted delivery of small molecule drugs is becoming increasingly important in personalized therapies, especially in cancer, brain disorders, and a wide variety of other diseases. However, effective means of spatial targeting and delivering high drug payloads in vivo are still lacking. Focused ultrasound combined with superheated phase-shift nanodroplets, which vaporize into microbubbles using heat and sound, are rapidly becoming a popular strategy for targeted drug delivery. Focused ultrasound can target deep tissue with excellent spatial precision and without using ionizing energy, thus can activate nanodroplets in circulation. One of the main limitations of this technology has been poor drug loading in the droplet core or the shell material. To address this need, we have developed a strategy to combine low-boiling point decafluorabutane and octafluoropropane (DFB and OFP) nanodroplets with drug-loaded liposomes, creating phase-changeable droplet-liposome clusters (PDLCs). We demonstrate a facile method of assembling submicron PDLCs with high drug-loading capacity on the droplet surface. Furthermore, we demonstrate that chemical tethering of liposomes in PDLCs enables a rapid release of their encapsulated cargo upon acoustic activation (>60% using OFP-based PDLCs). Rapid uncaging of small molecule drugs would make them immediately bioavailable in target tissue or promote better penetration in local tissue following intravascular release. PDLCs developed in this study can be used to deliver a wide variety of liposome-encapsulated therapeutics or imaging agents for multi-modal imaging applications. We also outline a strategy to deliver a surrogate encapsulated drug, fluorescein, to tumors in vivo using focused ultrasound energy and PDLCs.

## 1. Introduction

Active targeting of therapeutic molecules to a diseased tissue has long been an area of interest in pharmaceutical drug delivery. Researchers have developed and evaluated numerous passive or active targeting approaches in the past few decades [1,2,3]; however, the lack of a reliable targeting strategy persists in clinical applications. In particular, cancer therapy would benefit from better targeting approaches and more effective drug delivery due to the detrimental side effects of highly toxic chemotherapeutics. 

The most well-known targeting strategy in cancer therapies is the enhanced permeability and retention (EPR) effect, first introduced in 1986 by Matsumura and Maeda [4]. The pursuit of passive targeting approaches, hinging on the EPR effect for cancer therapy, gave rise to the need for nano-sized drug carriers in the 1990s. Since then, many studies have used circulating nanoparticles to deliver drug payloads, most of which relied on leaky vascular tissue in tumors to accumulate small drugs or drug carriers in the tumor [5,6,7,8]. Liposomal nanoparticles, in particular, have become more popular in cancer therapy following FDA approval of several formulations (namely, DOXIL^®^, DaunoXome^®^, Marqibo^®^, and Onivyde^®^). Such formulations have heralded significant promise for improving drug payload while reducing side-effects of toxic chemotherapies [6]. Despite promising results in animal models, passive targeting via EPR using liposomal drugs has only been partially successful in clinical trials [9,10,11]. This may be due to an exaggerated EPR effect in xenograft animal tumor models, which may not accurately reflect the tumor microenvironment in humans [12,13,14]. Thus, reliance on EPR alone may be insufficient for enhancing the delivery of liposomal drug carriers in certain cancers [15,16,17].

Limitations of EPR targeting necessitate the development of alternative strategies to achieve on-target drug delivery of nanomedicines. One such strategy is the release of small molecules from circulating nanocarriers in the tumor vascular space, which can then more easily penetrate a less permeable vascular endothelium. Rather than relying solely on the EPR effect, the driving force for penetration into the tissue is high local intravascular drug concentration, achieved through the rapid release of drugs from nanoparticle carriers. Subsequent uptake of the drug into tissue is mediated through a local concentration gradient driven Fickian diffusion process. This strategy has been explored using hyperthermia-mediated drug release from thermosensitive liposomes using compartment modeling [18], as well as in xenograft tumor models [19,20,21]. Clinical trials using this technology are currently ongoing using liposomal encapsulated doxorubicin [22]. A recent phase III clinical study using a heat sensitive liposome under the commercial name Thermodox™ was performed to treat unresectable hepatocellular carcinoma lesions [23]. Although Thermodox™ failed to meet FDA approval in this trial; treatment halted tumor growth and demonstrated improved survival in a subset of hepatocellular carcinoma (HCC) patients. A similar clinical trial has been performed using lyso-thermosensitive TARDOX and high-intensity focused ultrasound to target liver tumors [24,25]. While these studies have shown positive results, it has been noted that controlling drug release using thermally mediated processes can be somewhat challenging in deep tissue due to heat dissipation [26]. Furthermore, the time required to heat thermosensitive nanoparticles in fast-moving blood likely lowers the spatial resolution that can be achieved with targeting.

More rapid and efficient drug release from liposome carriers is possible using ultrasound responsive materials, specifically microbubbles and phase-shift contrast agents (PCAs). Microbubbles volumetrically change in the ultrasound (US) field, imparting shear forces on neighboring particles via acoustic streaming or inertial cavitation events [27]. They have been widely used for drug uncaging from various carriers, such as liposomes or hydrogels [28,29,30,31]. Klibanov et al. demonstrated partial drug release from liposome-conjugated microbubbles in vitro using calcein or thrombin-loaded liposomes. This group used focused ultrasound energy to inertially cavitate bubbles to trigger drug release [32]. More recently, Ozdas et al. demonstrated that low-intensity radiation-fragmentation pulses applied to liposome loaded-microbubbles could enhance targeted delivery of an encapsulated GABA_A_ receptor agonist (muscimol) without compromising the blood–brain barrier integrity [33]. However, short physiological half-lives of the bubbles, limited drug-loading capacity on the shell, and incomplete drug release are challenges that remain unaddressed. 

PCAs may be a better candidate for drug delivery applications due to their smaller size and longer-circulation times [34,35,36,37] and tunable vaporization thresholds [38] PCAs contain a superheated liquid perfluorocarbon (PFC) core. When exposed to heat or acoustic energy, the liquid PFC core vaporizes to form a gas microbubble [36,39,40,41]. This rapid liquid–gas phase transition can apply substantial shear forces to the surrounding boundaries [42,43,44], which makes them useful agents for permeabilizing tissue and promoting drug uptake [45]. One of the main advantages of PCAs is the ability to encapsulate PFC soluble compounds in the core, such as doxorubicin, for simultaneous imaging and therapy [46]. More recent research has shown that systemic drug release from the core of circulating nanodroplets can be achieved without inducing damage to surrounding blood vessels [47]. Although this exciting strategy has shown promising results [48], many drugs of interest are only minimally soluble in PFC. More efficient and versatile methods of drug loading or encapsulation would substantially improve on ultrasound-based drug delivery strategies.

PCAs are usually made from lipid, polymer [49], or protein shells [35] to stabilize the droplets and prevent them from coalescing. Sheeran et al., developed a method to produce droplets from the only microbubble formulation that is approved by the FDA which consists of a perfluorocarbon gas core stabilized by a phospholipid shell [50]. They showed that lipid shelled nanodroplets with decafluorobutane (DFB) or octafluoropropane (OPF) core are stable at physiological temperature due to the Laplace pressure exerted on droplets that prevent them from vaporizing [51]. This droplet formulation has been used for multiple applications including blood–brain barrier opening and drug delivery [45,52].

**Figure 1:**
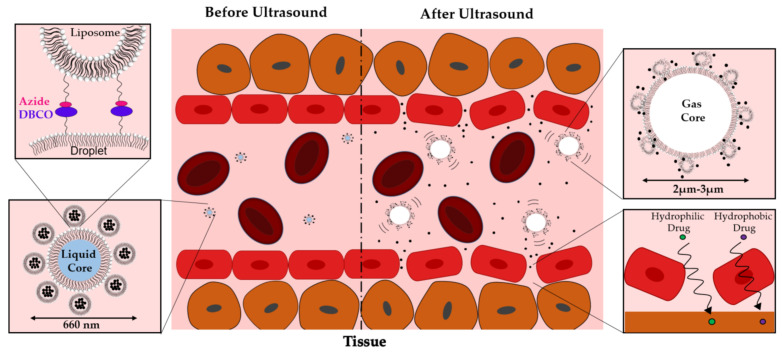
Schematic figure of phase changeable droplet-liposome clusters (PDLCs) activation in a vessel. Ultrasound induced droplet vaporization can both permeabilize vascular endothelium and disrupt liposomes within the construct to trigger intravascular drug release.

In the present work, we introduce the concept of nanoscale phase-changeable droplet-liposomes clusters (PDLCs) as vehicles for packaging and releasing small molecule drugs using focused ultrasound (Figure 1). Liposome nanoparticles, which are versatile drug carriers, are conjugated to the shell of DFB or OPF droplets using DBCO-Azide click chemistry to assemble the PDLCs. We hypothesized that the liposomes’ tethering to the droplet surface would significantly improve the release of encapsulated small molecules during droplet vaporization, thus making them immediately bioavailable. In this study, we demonstrate that conjugation of drug-loaded liposomes to the droplet surface can significantly enhance both drug loading on droplets and promote near-instantaneous release following a single ultrasound pulse sequence. We also demonstrate that using low boiling point DFB and OFP PDLC formulations can release 33% and 65% of liposome-encapsulated calcein, used as a surrogate drug. Finally, we outline a strategy for delivering small molecules to tumors in vivo using focused ultrasound. Based on our results, we conclude that PDLCs can be a useful tool for promoting small molecule drug delivery in a wide range of applications, including but not limited to cancer therapy. In this study, low boiling point lipid shelled droplets were used due to their compatibility and clinical relevance. 

## 2. Materials and Methods

### 2.1. Preparation of Lipid Films for PCA Droplet Production

Lipids were made by mixing of 1,2- distearoyl-sn-glycero-3-phosphocholine (DSPC, 790.16 MW), and *N*-(methylpolyoxyethylene oxycarbonyl)-1,2-distearoyl-sn-glycero-3-phosphoethanolamine (DSPE-PEG2000, 2805.97 MW (both from NOF Corporation, Tokyo, Japan) and 1,2-distearoyl-sn-glycero-3-phosphoethanolamine-n-[dibenzocyclooctyl (polyethylene glycol)-5000] (DSPE-PEG5000-DBCO,5000 Da, Nanosoft Polymers, Winston-Salem, NC, USA) using a molar ratio of 90:5:5 respectively (4 mg total lipid mass). Lipids were mixed by dissolving stock lipid powders in chloroform (Sigma-Aldrich, St. Louis, MO, USA) at 25 mg/mL and transferring them to a 2 mL serum vial (DWK Life Sciences, Millville, NJ, USA) using a glass syringe, (Hamilton Company, Reno, NV, USA) at the correct molar ratio. The chloroform was then evaporated using nitrogen gas; fully dried films were obtained by overnight vacuum desiccation. The following day, lipid films were hydrated with 2 mL of 1× phosphate buffered saline (PBS, Fisher Scientific, Waltham, MA, USA) containing 20% propane-1,2-diol (propylene glycol, 76.1 FW) (*v*/*v*) and 20% propane-1,2,3-triol (glycerol, 92.09 FW) (*v*/*v*). The lipid solution was heated to 65 °C in an isotemp heating block (FS Drybath Stdrd 4blk 100–120 V, Fisher Scientific, Waltham, MA, USA) and bath sonicated (1.9 L Ultrasonic Bath Sonicator, Fisher Scientific, Waltham, MA, USA) until the solution cleared and no large multilamellar lipid aggregates were observed (~15 min). After dispersing the lipids, a fluorescent dye, 1, 1′-dioctadecyl-3, 3, 3′, 3′- tetramethylindodicarbocyanine, 4-chloro benzene sulfonate (DiD) (Sigma Aldrich, Saint Louis, MO, USA) was added (0.2% dye to lipid ratio). The solution was sonicated again for an additional 10 min to mix DiD and lipids.

### 2.2. Preparation of PCA Droplets by Condensing Microbubbles

Perfluorocarbon droplets were generated using a microbubble condensation method outlined by Sheeran et al. [41]. Precursor microbubbles were synthesized by vial mixing, as described by Chen et al. [53]. Either DFB or OFP gas (Fluoromed, Round Rock, TX, USA) was introduced into the headspace of the vial, after which mixing was performed by a Vial mix dental amalgamator (Bristol-Myers Squibb Medical Imaging New York, NY, USA) for 45 s. Microbubble solutions were cooled to −17 °C in a bath of 2-methyl butane (Sigma Aldrich, St. Louis, MO, USA). After cooling, a 27 G needle was used to pierce the vial cap and apply atmospheric pressure to the cooled microbubbles to cause condensation. Droplets were separated from liposomes by centrifugation using a benchtop centrifuge (Sprout, Heathrow Scientific, Vernon Hills, IL, USA) at 2000× *g* for 10 min and resuspended in solution. This process was repeated three times to remove liposomes.

### 2.3. Preparation of Fluorescent Liposomes for PDLC Formation 

The liposomes for PDLC preparation were made by film hydration and bath sonication. Briefly, lipid films were made using the same protocol outlined above. A mixture of DSPC, DSPE-PEG2000, and 1,2-distearoyl-sn-glycero-3-phosphoethanolamine-*N*-[azido(polyethylene glycol)-5000] (DSPE-PEG2000-Azide, 5000 Da) (Nanosoft Polymers, Winston-Salem, NC, USA) in a molar ratio of 90:5:5, respectively, was prepared from lipid stock solutions using 16 mg of total lipid. Nitrogen gas was used to evaporate chloroform. The sample was further dried in a vacuum chamber overnight. The lipid film was hydrated (8 mg/mL) using 2 mL of 1X PBS solution and heated to 65 °C for 15 min. The solution was bath sonicated for 30 min to dissolve the lipids. Experiments requiring fluorescent liposomes incorporated a 3, 3′-Dioctadecyloxa carbocyanine perchlorate (DiO) labeled into the shell at 0.2% dye to lipid ratio, then sonicated again for an additional 10 min.

### 2.4. Preparation of Calcein-Loaded Liposomes for PDLC Formation

For drug encapsulation and release experiments, calcein was used as a surrogate small molecule drug. We used the established calcein encapsulation method in the literature [54,55]. Briefly, A 50 mM calcein (MP Biomedicals, LLC, Solon, OH, USA) solution was made in ultra-pure water. NaCl salt (Fisher Chemical, Fair Lawn, NJ, USA) was added to the solution to adjust the osmolarity (~320 mOsm). The pH was measured using a Starter 2100 pH Bench Meter and STMicro5 pH probe, Ohaus Corporation (Parsippany, NJ, USA), and adjusted to 8.5 by adding a concentrated hydrochloric acid solution (Sigma-Aldrich, St. Louis, MO, USA) dropwise.

Lipid films for calcein-loaded liposome preparation were made the same way as described in the previous section. Lipid films were hydrated using 2 mL of 50 mM calcein solution (8 mg/mL) and heated to 65 °C on a heating block for 15 min. The solution was bath sonicated for 30 min to generate unilaminar vesicles. After sonication, the sample was subjected to five freeze–thaw cycles to maximize the loading of calcein in the liposome core. During the freezing phase, samples were placed on dry ice for 3 min. During the thawing stage, they were maintained at room temperature for 8 min. In order to wash non-encapsulated calcein from the solution, gel filtration was performed using spin columns and Sephadex G-50 Medium (GE Healthcare, Uppsala, Sweden). The solution was filtered two times at 1000× *g* for 2 min at room temperature using a 5806R centrifuge Eppendorf (Hamburg, Germany). 

### 2.5. Formation of PDLCs from PCA Nanodroplets and Liposomes

Liposome loading of droplets was performed using DBCO-Azide click chemistry. DBCO-coated droplets were added dropwise to Azide-coated liposomes at a 10:1 liposome-lipid:droplet-lipid ratio. The total amount of the lipid in the droplet samples was estimated based on the fluorescence intensity of DiD in the droplet shell and known standard curves. The ratio of 10:1 was based on a previous study by Hall et al., using a similar microbubble clustering approach [56]. The amount of total lipid in PCAs and liposomes was estimated based on fluorescent intensities of DiO or DiD in the liposomes and droplets and known standard curves. The mixture was incubated and mixed using a rotatory mixer (RotoFlex R2000, Argos Technologies, Vernon Hills, IL, USA) overnight at 4 °C to allow the reaction to proceed. After incubation, the resulting PDLCs were washed by centrifugation (400 RCF, 25 min, 4 °C) to remove unbound liposomes. The PDLCs were used immediately for characterization and drug release studies.

Controls in the experiment used Sodium azide (Sigma-Aldrich, St. Louis, MO, USA) to block the DBCO—Azide interaction before forming PDLCs. Sodium azide solution was made using deionized water as the solvent (10 mg/mL). DBCO droplets were incubated with Sodium azide solution (1:1000 DBCO to Azide ratio) in the rotatory mixer for 5 min to optimally block DBCO functional groups on the droplets and prevent binding. 

### 2.6. Characterization of PDLCs Using Microscopy

The PDLCs were characterized by brightfield and fluorescence microscopy with either a BX50 Upright Microscope (ACH 60X/0.80 ∞/0.17 objective) or an IX70 Inverted Microscope (ACH 60X/0.80 ∞/0.17 objective) with a 100 W High-Pressure Mercury Burner, Olympus (Waltham, MA, USA). Diluted samples of PDLCs were pipetted onto 25 × 75 × 1 mm microscope slides and placed under a glass coverslip (Fisher Scientific, Waltham, MA, USA) in preparation for imaging. The appropriate filters were used to separate DiO and DiD fluorescence. Microscopy pictures were acquired by a Rolera Bolt CMOS QImaging Camera (Surrey, BC, Canada). Images for both the PDLCs and the DBCO blocked negative control groups were acquired from three independent samples (*n* = 3) using ten images per sample.

### 2.7. Characterization of PDLCs Using Dynamic Light Scattering

Malvern Zetasizer Nano ZS dynamic light scattering (Cambridge, UK) was used to measure the size distribution of the PDLCs in a 3 mL polystyrene cuvette (Fisherbrand, Waltham, MA, USA). Three different groups, (1) liposomes alone, (2) droplets alone, and (3) PDLCs, were characterized to determine the effect of chemical conjugation on size distribution. The concentration of droplets and liposomes in each group was estimated using the fluorescence intensities of the DiD and DiO in the droplets and liposomes, respectively. The concentration of lipids was matched prior to analyzing each sample. The reported size is the mean value of three independent measurements. 

### 2.8. Quantification of Calcein Loading in PDLCs 

Calcein loading in the PDLCs was measured using the fluorescent calcein signal after separating unconjugated liposomes. Polyethylene glycol tert-octylphenyl ether (Triton X-100) (Sigma-Aldrich, St. Louis, MO, USA) was used to destabilize intact liposomes of the PDLCs and release encapsulated calcein for quantification. Then, 100 µL of concentrated PDLCs solution was diluted 20-fold with PBS containing 10% *v*/*v* Triton X-100. Free calcein was measured using a plate reader (Synergy H4 Biotek, Winooski, VT, USA) at 490 nm excitation, and 520 nm emission wavelengths. The total calcein loading was calculated using a standard curve with known calcein concentrations. All data were measured in triplicate. Azide-blocked DBCO droplets and calcein-loaded liposomes were used as negative controls to determine the background fluorescent signal.

### 2.9. Drug Release from PDLCs during Ultrasound Application In Vitro

Calcein release was measured using a custom made acoustic chamber. The acrylic chamber was filled with water and heated to the desired temperature (37 °C for OFP PDLCs or 45 °C for DFB PDLCs) using an immersion heater (Ulanet 324, Columbus, IN, USA). The temperature of the bath was monitored using a digital temperature probe (Fisherbrand Traceable Digital Thermometer, Waltham, MA, USA) and maintained at a constant level for the duration of the experiment. A focused ultrasound (US) transducer (H-131 Sonic Concepts, 1.1 MHz, Bothell, WA, USA) was used to activate the PDLCs. An ultrasound pulse-echo system (5072PR Olympus, Shinjuku, Tokyo, Japan) was used to create a 5-pulse train at 100 Hz to drive the transducer, producing a peak negative pressure (PNP) of 1.55 MPa (measured by a needle hydrophone, HNC-0100, Ondacorp, Sunnyvale, CA, USA). The sample was insonified for a total of 10 min. A focused hydrophone (Y-107, Sonic Concepts, Bothell, WA, USA) was used to listen for the acoustic response of the activated droplets. The transducer and the hydrophone were positioned at a 90-degree angle. To align the transducer and the hydrophone so that both foci would coincide on the same spot, a microscope slide held by a custom holder was used to reflect the output of the transducer to the hydrophone. The position of the hydrophone was adjusted to capture the reflected signal. After alignment, the microscope slide holder was removed. Next, 1 mL of PDLC solution was slowly transferred into a small pipette bulb (Fisherbrand, Waltham, MA, USA) secured by a custom-designed, 3D printed holder and placed in the water bath where the hydrophone and the transducer were aligned. The sample was left in the water bath for 5 min to equilibrate to temperature (37 °C for OFP Clusters or 45 °C for DFB clusters) before the start of sonication. The sample was mixed using a polypropylene gel loading pipette tip (Fisherbrand, Waltham, MA, USA) every 2 min during sonication. Calcein release was measured before starting sonication, and at 5 min and 10 min after sonication using the plate reader (Synergy H4 Biotek, Winooski, VT, USA). Two samples (100 µL each) were removed from the chamber. One sample was diluted in standard 1X PBS. The other sample was diluted with 7.4 pH-adjusted 10% *v*/*v* Triton 100X (Sigma-Aldrich, St. Louis, MO, USA) to release all calcein entrapped within the liposomes. The difference in fluorescence intensities was used to calculate the percentage of calcein released by using Equation (1), similar to methods used by Maherani et al. [57].

%Release = (F_son_ − F_i_)/(F_total_ − F_i_) × 100 (1)

F_son_ is the fluorescence intensity of the sonicated sample, F_i_ is the initial fluorescence of the sample before insonifying and F_total_ is fluorescence after adding Triton and releasing all of the entrapped calcein. The vaporization signals detected by the hydrophone and the transducer were used to verify the completion of droplet vaporization. Control experiments were performed with liposomes only, PDLCs formed with DBCO blocking, and co-mixed droplets and liposomes. All experiments were performed in triplicate.

### 2.10. Drug Release from PDLCs in Flow

A custom chamber was developed to visualize drug release in a capillary tube, similar to the setup outlined by others [30,57]. A water bath contained within an acrylic chamber was placed onto an Olympus 1X-71 inverted microscope and heated to 45 °C using an immersion heater (Ulanet 324, Columbus, IN, USA). The PDLC solution was infused through a 200 μm-diameter cellulose capillary tube (Spectrum Laboratories, Irving, TX, USA) inside the bath, positioned on the focal plane of a 60X water-immersion objective (Olympus, Shinjuku, Tokyo, Japan). A 1.1 MHz ultrasound transducer (H-131 Sonic Concepts, Bothell, WA, USA) with a focal length of 35 mm was used to apply ultrasound pulses to PDLCs in the microscope’s field of view. The ultrasound transducer was driven by an arbitrary waveform generator (Picoscope5000 with AWG; Cambridgeshire, UK) with the RF signal amplified by a linear amplifier (ENI 2100L; Rochester, NY, USA). The PNP of the ultrasound pulses was measured using a needle hydrophone (HNC-0100; Ondacorp; Sunnyvale, CA, USA) attached to an oscilloscope (Picoscope5000). A single ultrasound pulse train (300 pulses total) was applied at 1.85 MPa PNP. A digital QImaging Optimos CCD camera (Surrey, BC, Canada) mounted on the inverted scope (1280 × 1280 resolution; 100 frames per second) was used to take the images. Application of ultrasound and image capture were synchronized using a custom LabVIEW program and data acquisition board (Arduino UNO) to delay the ultrasound pulse by 10 ms following the camera acquisition trigger. Digital images were displayed and processed using NIH ImageJ software and Micromanager acquisition software. Fluorescent images were acquired using a 100 W High Pressure Mercury Burner, Olympus (Shinjuku, Tokyo, Japan).

### 2.11. Droplet Vaporization In Vitro Using Therapeutic Ultrasound Application 

To visualize vaporization of OFP PDLCs following application of focused ultrasound, a commercial handheld therapeutic transducer (SoundCare Plus, Austin, TX, USA) coupled to a custom milled lens-and-cone was focused onto an acoustically transparent pipette bulb filled with 1 mL of 1× PBS that was submerged in a water bath maintained at 37 °C. B-mode imaging was performed simultaneously using an Acuson Sequoia 512 ultrasonography system (Siemens Medical Solutions, Erlangen, Germany), equipped with a linear 15L8 transducer. The focused ultrasound probe was powered at an intensity of 3 W/cm^2^ (corresponding to 2 MPa peak negative pressure in the focal zone) and a 10% duty cycle to insonify droplets as they were injected at 4 °C temperature into the pipette bulb. A T-type thermocouple probe (ADInstruments, Colorado Springs, CO, USA) was placed inside of the pipette bulb to monitor the PDLC solution temperature. Data was recorded using proprietary LabVIEW software. The sonication process started after PDLC solution reached 30 °C in which the transducer was on/off for 5 s each until all vaporized droplets disappeared from the field of view. To visualize the vaporization threshold, proper regions of interest (ROIs) were selected and the pixel intensities were measured in the ROI. 

### 2.12. Fluorescein Uptake in Matrigel™ Plugs In Vivo

In vivo drug delivery studies were performed with PDLCs in Matrigel™ plug “mock” tumors. The Matrigel™ was implanted in Female CD-1 mice (Charles River, Wilmington, MA) as outlined previously by Bellary et al. [58]. Briefly, 10 mL of frozen Matrigel™ was thawed overnight on ice. Then, 12 μg human recombinant basic fibroblast growth factor (bFGF, Stemgent, Beltsville, MD, USA) and 350 μg heparin were added to 10 mL Matrigel™ to stimulate blood vessel ingrowth after implantation. For each mouse, 1 mL of Matrigel™ was loaded into chilled 1 mL BD syringes using chilled 16-gauge needles. After loading, the needle was replaced with a 27-gauge needle and kept on ice until animals were prepared. No exogenous cells (tumor or endothelial) were used in Matrigel™ studies. All animal experiments were approved by the UT Dallas IACUC. Mice were anesthetized with 2–3% isoflurane (Vedco, St. Joseph, MO, USA) and restrained in the prone position. A shaved and disinfected region above the left kidney received 1 mL of chilled Matrigel™ solution subcutaneously, creating a spherical plug (~1 mm^3^).

For this experiment, OFP fluorescein-loaded PDLCs were made (instead of calcein) as described previously. Briefly, 50 mM Fluorescein stock solution was made from fluorescein powder (Fluorescein sodium salt, Sigma-Aldrich, St. Louis, MO, USA). Fluorescein-loaded, DiD labeled liposomes were made similar to calcein-loaded liposomes and went through 5 cycles of freeze–thaw and 2 spin gel filtrations using Sephadex 50 gel. PDLCs were made as discussed previously using DiD labeled OFP droplets and fluorescein-loaded liposomes. 

PDLCs were injected intravenously while keeping the syringe on a frozen ice pack. 30 s following the injection, implanted Matrigels™ were insonified (3 W/cm^2^) at 10% duty cycle by a therapeutic transducer as described earlier. Sonication was applied for 8 min, turning the transducer on/off every 5 s to allow reflow of fresh PDLCs. Matrigel™ plugs were surgically excised for ex vivo analysis 20 min after PDLC injection, as described by Bellary et al. [58]. Excised plugs were frozen using dry ice-cooled 2-methyl butane (Sigma Aldrich, St. Louis, MO, USA) and kept in tubes in freezer (−80 °C). Then, 24 h after freezing, the plugs were sectioned for imaging, and DAPI containing vectashield mounting medium (Vector Laboratories, Inc. Burlingame, CA, USA) was used to dye cell nuclei. Images were taken using an Olympus VS120 Virtual Slide Microscope. Data and images were analyzed using Olympus VS120 Virtual Slide Microscope. Fluorescein uptake in Matrigels™ treated with PDLCs was compared to Matrigels™ treated with liposomes alone and untreated Matrigels™. A total of 2 mg of fluorescein (encapsulated in liposomes) was injected into each mouse.

## 3. Results and Discussion

### 3.1. Characterization of PDLCs via Microscopy and Fluorescence Intensity

**Figure 2:**
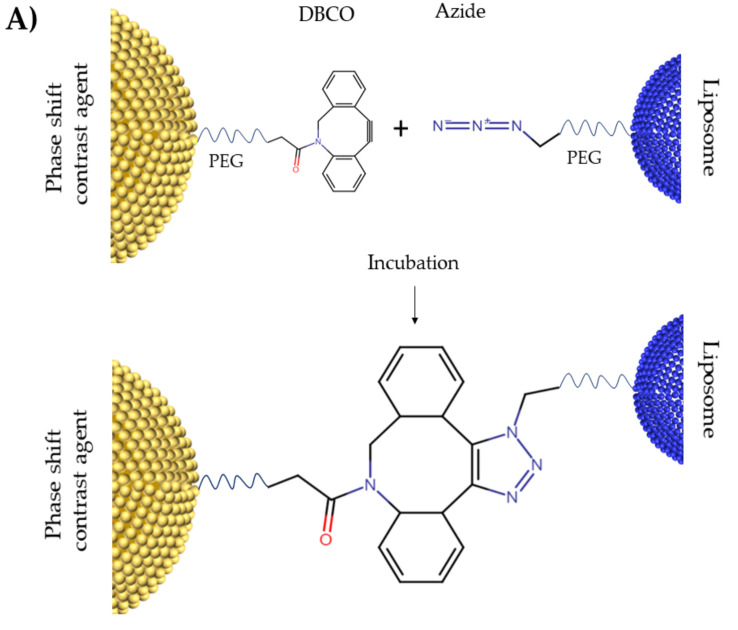

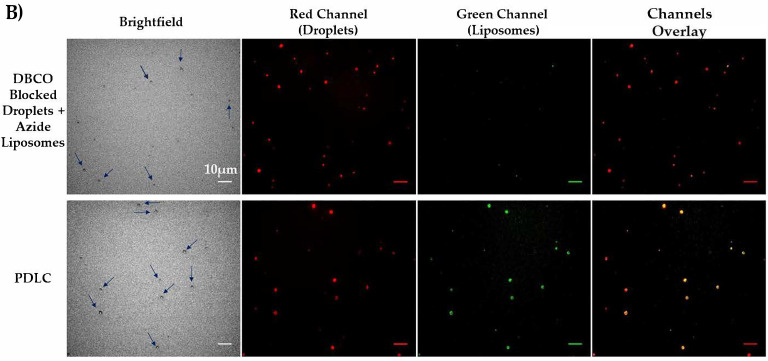
(**A**) Schematic figure for DBCO-Azide click chemistry. (**B**) Fluorescent Microscopy of PDLCs after washing the unbound liposomes. Phase changeable droplets are dyed with DiD (red) and liposomes are dyed with DiO (green). There are no liposomes bound to the droplets in the top row since DBCO binding groups on droplets’ shell in this sample are blocked with sodium azide. Colocalization of green liposomes and red droplets (yellow) in the PDLC sample shows the clustering of droplets and liposomes. All scale bars show 10 µm.

Our initial aim of the study was to demonstrate a facile method of producing PDLCs using a single-step strain-promoted [3 + 2] azide-alkyne cycloaddition (SPAAC) click chemistry approach, motivated by Slagle et al. [59]. Brightfield and fluorescent microscopy were used to visualize PDLCs and assess the binding efficiency of DBCO-Azide click chemistry 24 h after incubation (Figure 2). Liposome nanoparticles themselves cannot be seen using brightfield microscopy due to their small size and low index of light refraction. Therefore, no qualitative difference in negative control and PDLC sample was observed in brightfield microscopy. However, fluorescent microscopy showed that the co-localization of DiO labeled liposomes (green) and DiD labeled droplets (red) occurs in PDLCs sample, but not in the DBCO-blocked negative control (Figure 2B), indicating that the binding of liposomes occurs primarily due to DBCO-Azide chemistry.

**Figure 3:**
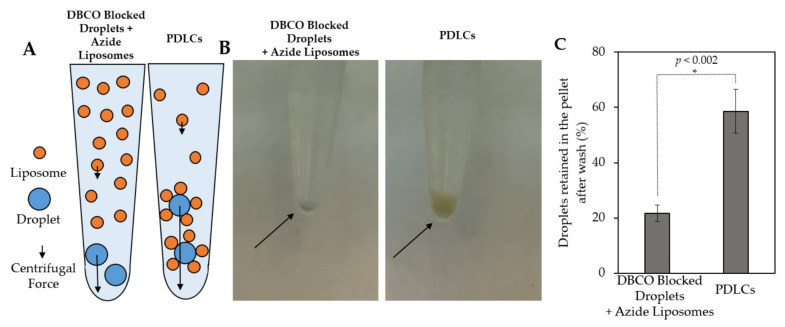
Centrifugation of calcein-loaded PDLCs. (**A**) Schematic figure of centrifugal forces applied to each particle during centrifugation. The magnitude of the centrifugal force has a direct relation with the mass of the particle. (**B**) Samples after centrifugation and removal of the supernatant. The absence of conjugation in DBCO blocked droplets causes the washing of the unbound liposomes. The orange color of the pellet in the PDLC sample is indicative of the existence of quenched calcein in the liposomes that are bound to droplets and retained in the pellet. (**C**) Percent of droplets retained in the pellet after three centrifugations, as determined by reduction in DiD intensity. Droplets in the PDLC sample carry liposomes and are consequently heavier so they experience higher centrifugal forces. The difference is statistically significant (*p* < 0.002) and is another indication of tethering droplets to liposomes.

DiO-labeled Azide-Liposomes were subsequently replaced with calcein-loaded liposomes. Following 24 h incubation of droplets and liposomes, the samples were washed by centrifugation, as performed for microscopy experiments. After three washing steps, the PDLC samples consistently had larger pellets (Figure 3). The PDLC pellets exhibited an orange color from the quenched calcein inside of liposomes while the negative control showed smaller pellets with a light blue color from the DiD. The difference in pellet size is indicative of a larger mass in the PDLC sample in comparison with the DBCO-blocked negative control, wherein no calcein liposomes were collected. The number of droplets collected was quantitatively evaluated using the DiD fluorescence signal from the droplets to estimate the recovered percentage of lipid after washing compared to the signal intensity in the pre-washed samples (Figure 3C). Our results indicate that both liposomes and droplet recovery is higher in the PDLC sample compared to the blocked unconjugated liposomes and droplets. It is possible that conjugation of liposomes to droplets would create a less neutrally buoyant particle that collects more efficiently during the centrifugation process.

Overall, our microscopy and fluorescence analysis results (Figure 2 and Figure 3) show that incorporation of DBCO-PEG5K-DSPE into the droplets shell easily promotes conjugation with drug-loaded liposomes containing Azide-PEG5K-DSPE. At 24 h after mixing droplets and liposomes, microscopy shows that 100% of the DBCO droplets observed were attached to liposomes and resulting PDLCs were still in submicron size ranges. While we expect that the DBCO-PEG-lipids on the droplet are fully saturated at 24 h, this was not determined in this study. It may be possible to increase drug loading with increased liposome concentrations and longer incubation periods. However, we determined the methodology for PDLC formulation to be adequate for demonstrating drug uncaging from vaporization. It is worth mentioning that some liquid PFC may be lost in the conjugation process due to either spontaneous vaporization or PFC leakage from the core. We did not verify PFC content after conjugation, however previous publications have shown this droplet formulation to be highly stable [50] and was originally developed to prolong the shelf-life of perfluorocarbon bubble [60]. We also observed a high degree of vaporization at consistent thresholds, thus the PFC content in droplet core is considered adequate after formulation.

### 3.2. Characterization Using Dynamic Light Scattering (DLS)

**Figure 4:**
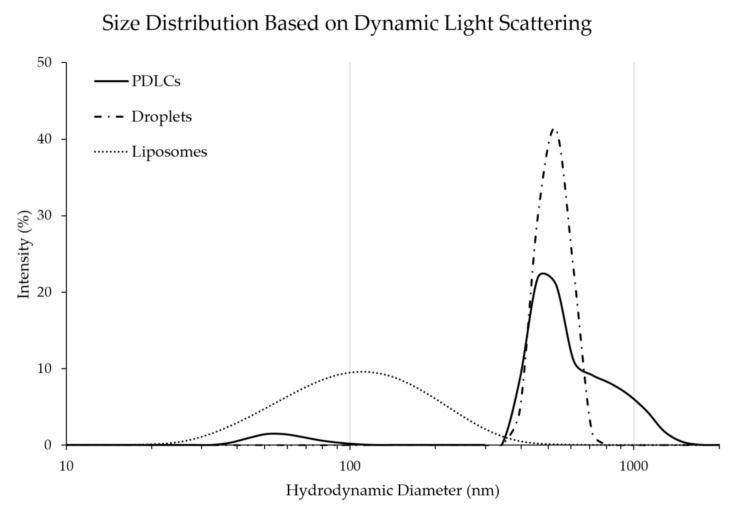
The size distribution of droplets, liposomes, and PDLCs. The clustering of droplets and liposomes increases the overall median diameter of the particles. Distributions are averaged from three sample measurements.

DLS showed that the liposomes had a median diameter of 120 nm +/− (6 nm), whereas the median diameter of droplets was 547 nm +/− (26 nm). PDLC exhibited two peaks in the size distribution. The larger peak is presumed to be from the PDLC and showed an increase in the median diameter to 652 nm +/− (120 nm) compared to the droplets alone, indicating the formation of larger aggregates (Figure 4). This could potentially be due to the crosslinking of PDLCs during formation. The smaller peak at 63 nm is presumed to be unremoved liposomes remaining in the pellet after three washing steps. 

The DLS measurements of PDLCs showed a high degree of polydispersity in size distribution (Figure 4). We expect that this is due to the wide distribution of starting liposome sizes rather than variability in the starting droplet sizes. A majority of the PDLCs did not appear to change in diameter compared to droplets. However, this may be due to the interactions of droplets in the dynamic light scattering machine, which interpolates size from the Brownian motion of particles [61]. The density of the perfluorocarbon is ~10-times greater than the liposome and thus may largely be the dominant component of the PDLC that determines size in this assay. The PDLCs do exhibit a larger distinct shoulder on the larger diameter side. This may be due to some droplet crosslinking (although this was not apparent in qualitative microscopy analysis) or conjugation of larger liposomes to the droplet surface which may affect the Brownian motion. However, most PDLCs stayed in the nano-range (>92%) and would be suitable for in vivo injections. We expect that the size distribution of the PDLCs can be narrowed by using more monodispersed droplets and liposomes precursors. 

### 3.3. Calcein Loading in PDLCs

The total amount of calcein loading in PDLCs was measured using a fluorescent plate reader. Negative controls, using (1) Sodium azide-blocked DBCO droplets mixed with liposomes; and (2) liposomes alone, were used to account for residual liposomes after washing and evaluate non-specific adsorption. The total amount of loaded calcein in PDLCs was measured to be 2.8 µM (+/−0.14), which is a ~7-fold increase in calcein loading compared to Sodium azide-blocked DBCO droplets. One-way ANOVA showed a significant difference between the groups (*p* < 0.0001). Post hoc *t*-tests showed significance between the PDLC group and both of the other groups (*p* < 0.0001). 

**Figure 5:**
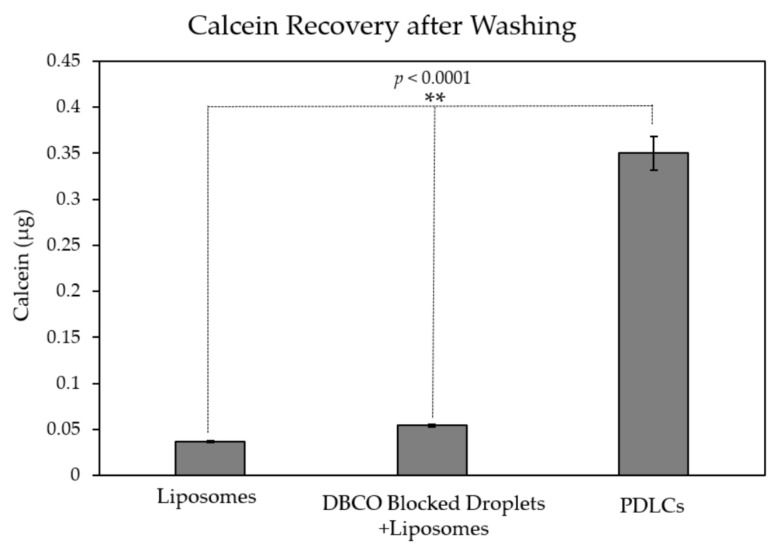
Calcein recovery after washing. Increased calcein recovery is observed in PDLC samples compared to liposomes (** *p* < 0.0001). DBCO blocking of droplets before clustering shows little non-specific binding indicated by the minimal increase in calcein recovery compared to liposomes alone.

The presence of untethered liposomes was observed in all samples (Figure 5). The small fraction of liposomes that remains in the pellet is likely due to large liposomes that sink during centrifugation. The untethered liposomes thus cannot be removed by centrifugation and may account for nearly 10% of the calcein in PDLC samples.

### 3.4. Calcein Release from PDLCs during Ultrasound Application In Vitro

**Figure 6:**
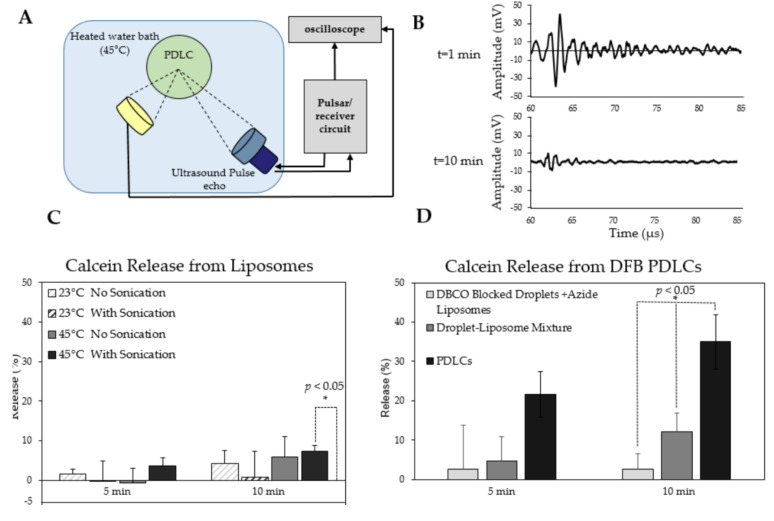
Calcein release from phase changeable droplet-liposome clusters (PDLCs) after 5 and 10 min of sonication (**A**) schematic illustration of the acoustic chamber setup used for measuring calcein release from PDLCs. (**B**) vaporization acoustic signals during and after vaporization used for visualizing the completion of droplet vaporization. (**C**) Effect of sonication and temperature on release from liposomes alone (no droplets). Only sonication at 45 °C showed a statistically significant release above zero (* *p* < 0.05). (**D**) Calcein release following US exposure. Release from PDLCs was significantly higher than any other group after 10 min (* *p* < 0.05). Release after 5 min was higher than the negative controls but not significantly. *N* = 3 per group.

Calcein release from PDLCs was measured in vitro using a co-aligned focused transducer and hydrophone (Figure 6A). The droplets’ acoustic response, detected by the hydrophone and the transducer, subsided by the 10 min mark, indicating complete activation of the PDLCs (Figure 6B). Calcein release from liposomes was first measured without droplets to determine their stability after 5 and 10 min of sonication (Figure 6C). Overall, the liposomes appeared stable with little difference at either 23 °C or 45 °C with or without US exposure. No significant release of calcein was observed at room temperature with or without sonication. No release was seen at 45 °C without sonication, but some calcein release was observed with sonication at 45 °C (~ 8% calcein release, *p* < 0.05). However, the total amount of calcein leakage was substantially lower than release from PDLC vaporization as described below. 

Calcein release from PDLCs was measured following ultrasound application at 45 °C (Figure 6D), which was the temperature at which vaporization was consistently observed using passive cavitaion detection. PDLC samples showed a 35% (+/−6.8) release after 10 min of insonification, which was significantly higher compared to DBCO-blocked droplets and Azide liposomes or co-mixed droplets and liposomes (*p* < 0.05). Total calcein release from all groups was corrected using the spontaneous calcein leakage from liposomes alone at 45 °C. One-way ANOVA showed statistical differences between the three groups (*p* < 0.05). Further *t*-tests between each group showed significance between the PDLC group and both other groups (*p* < 0.05). The *t*-test between the two negative controls showed no significance (*p* > 0.05). This data indicates the release of the calcein is significantly improved by the chemical tethering of the liposomes to droplets. It was also noted earlier that unconjugated liposomes in the PDLC sample may affect both calcein loading and calcein release calculations. Alternatively, it is possible that many of the droplets may not activate at the specific pressure and temperature chosen. In later experiments, more complete activation could be observed using lower boiling point OFP as the droplet core.

It is important to note that the 10 min time-scale used in this experiment does not reflect the true release kinetics of encapsulated drug from the PDLC upon vaporization, but rather the time required to activate all droplets in the chamber completely. It is expected that the release of calcein from each PDLC occurs instantaneously during the vaporization process, which is demonstrated in the subsequent experiment.

The effect of conjugation of liposomes on droplet vaporization was also assessed in this study. Previous studies have suggested that aggregation of lipid shelled droplets can change the vaporization behavior [62]. The vaporization threshold of droplets were measured when bound to liposomes and were compared to a mixture of unbound droplets and liposomes. This study shows that conjugation of droplets to liposomes did not cause any change in the activation threshold of the droplets (Supplementary Materials, Figure S1).

### 3.5. Calcein Release from PDLCs in Flow

**Figure 7:**
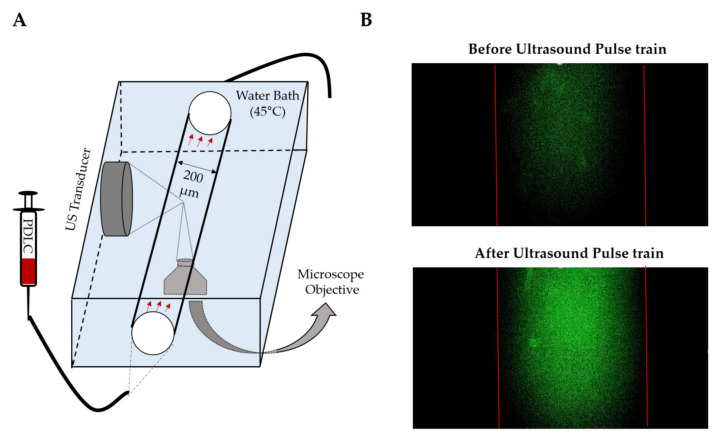
Calcein release from decafluorobutane (DFB) PDLCs inside of a capillary after a single pulse train (300 cycles,1.85MPa, 1.1 MHz) (**A**) Schematic setup for visualizing calcein release in a capillary tube. (**B**) Fluorescent images of PDLCs before and after US exposure. An instant increase of fluorescence in the green channel shows the instant release of calcein inside of the capillary tube. This experiment was repeated on three independent samples.

A flow phantom was used to visually observe the vaporization and calcein release from PDLCs using brightfield and fluorescent microscopy (Figure 7A). Immediately after a single ultrasound pulse train, an increase in fluorescence intensity was observed (Figure 7B). As evident in the videos, calcein fluorescence faded away after the immediate release as the calcein molecules diffuse out of the field of view or were photobleached (Supplementary Video S1). After vaporization, gas bubbles were observed that rapidly coalesce, indicating that the resulting microbubbles are less stable than pre-formed microbubbles (Supplementary Video S1). This is likely due to the inability to incorporate additional lipids into the shell when vaporized [63]. Overall, these results demonstrate that rapid release of small molecules can be achieved to make drugs bioavailable in circulation or possibly enhance intratumoral drug penetration by enhancing concentration mediated diffusion. We expect that optimizing ultrasound pulsing can maximize the local concentration of the drug in the focal region. However, we did not measure the total release of calcein in this part of the study. 

### 3.6. Calcein Release from Low-Boiling Point OFP-PDLCs 

Until this point, PDLCs were fabricated using DFB microbubbles condensed into droplets. One of the significant limitations of this formulation is that temperatures of at least 45 °C were needed to observe adequate vaporization from ultrasound exposure in our studies. Reducing and controlling the vaporization threshold is one of the major goals concerning acoustic droplet vaporization (ADV), especially for clinical drug delivery applications [50]. Other groups have shown that OFP can significantly reduce activation temperatures and vaporization thresholds [44,50]. We used a similar approach in our studies to lower the vaporization temperature and acoustic threshold of PDLCs using condensed OFP microbubbles to form the droplet core. 

**Figure 8:**
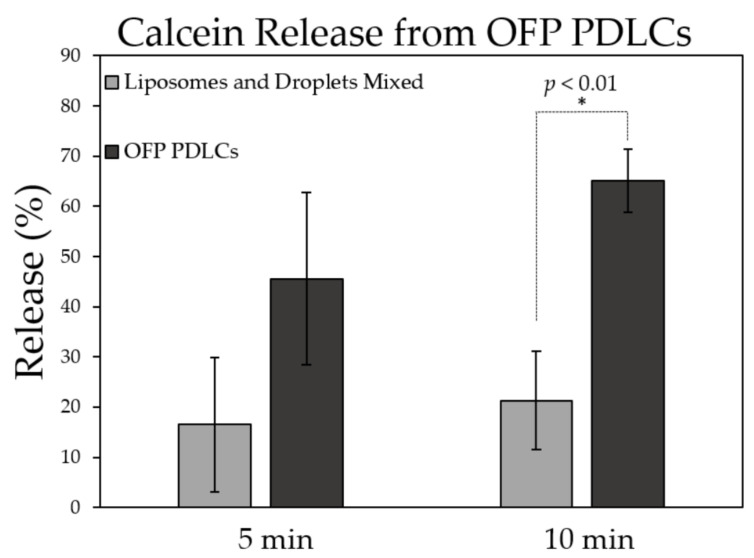
Calcein release from octafluoropropane (OFP) PDLCs during ultrasound exposure at 37 °C. Calcein release was significantly higher after 10 min US exposure (* *p* < 0.01) compared to equivalent concentrations of droplets and liposomes mixed in solution. *N* = 3 per group.

OFP-PDLCs were prepared as described in the methods section, similar to DFB PDLCs. Although the yield of PDLC production was much lower than DFB PDLCs (~10 times lower due to more spontaneous vaporization), the release rate for OFP PDLCs was 65% (+/−6.2) (Figure 8), which was higher than DFB PDLCs (65% vs. 35%). A *t*-test between the two groups showed a significant increase in release when OFP droplets are bound to liposomes (*p* < 0.01).

While DFB-PDLCs have a higher boiling-point and longer shelf-life compared to OFP-PDLCs, vaporization of OFP was more efficient at 37 °C due to the lower activation threshold of the perfluorocarbon. However, OFP droplets appeared to undergo spontaneous droplet vaporization and needed to be used immediately after production. The volatile nature of OFP PDLCs makes them challenging to handle and difficult to use for experiments; however, they are likely necessary for clinical translation in non-hyperthermia based applications. In the final experiments, we demonstrate how OFP-based PDLCs can be activated with a therapeutic transducer and deliver drugs to tumors in vivo. 

### 3.7. Droplet Vaporization In Vitro Using Therapeutic Ultrasound Application 

To further test the activation of OFP-PDLC and their potential use for image-guided drug delivery, we used a custom therapeutic focused ultrasound transducer in combination with a clinical scanner to test the feasibility of vaporizing OFP-based PDLCs. Droplets will not produce contrast in a B-mode imaging due to the incompressible nature of PFC liquid in the core. However, vaporization events will dislocate the aqueous medium and produce a detectable acoustic signal. Further sonication will also produce contrast, as the formed bubbles will oscillate volumetrically in an acoustic field. 

**Figure 9:**
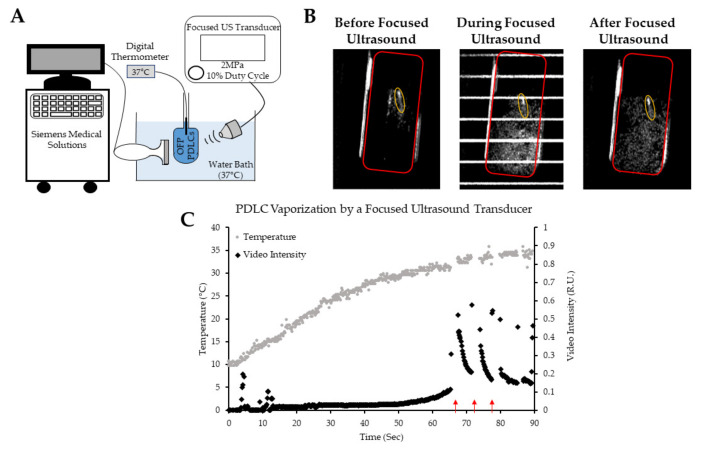
Visualizing focused ultrasound-induced octafluoropropane droplet vaporization using a clinical scanner. (**A**) Schematic of the setup used for acquiring B-mode images to monitor OFP droplet vaporization. (**B**) B-mode images showing the vaporization of OFP droplets before, during, and after focused ultrasound application. The region of interests (ROIs) used for intensity quantifications are shown. The temperature probe inside of the tube can be seen in the B-mode images and is shown by the yellow ellipse. (**C**) Video intensity within the chamber (ROIs showed in red). The red arrows show sonication starting points.

When OFP-PDLCs were placed in a 37 °C water bath, some spontaneous or easily triggered vaporization events were observed using B-mode imaging (Figure 9B) before focused ultrasound was applied. However, the intensity of vaporization signals increased drastically after focused US exposure, suggesting that a large population of OFP droplets vaporized rapidly upon excitation with focused US. The bubbles resulted from droplet vaporization were destroyed following further sonication. (Supplementary Video S2).

Figure 9C shows the vaporization of OFP PDLCs when exposed to ultrasound as the PDLCs warm to bath temperature (37 °C). No vaporization is observed until the PDLC solution reached ~30 °C, after which spontaneous activation appeared to be more frequent. Focused ultrasound was then applied, triggering a rapid increase in video intensity from ultrasound-triggered PDLC activation. The increase in video intensity was reduced after each subsequent sonication cycle, suggesting PDLCs were rapidly being depleted. After nearly 30 s, no signal change was observed after each sonication cycle. Overall, this experiment demonstrated that PDLCs should be introduced cold to minimize spontaneous activation and that focused ultrasound can be used to activate and release OFP-PDLCs at physiologically relevant temperatures and pressures. It is also possible to activate droplets by increasing the output on the clinical imaging machine itself, as demonstrated by De Gracia Lux et al. [64]; however, focused ultrasound can provide much better spatial control of droplet vaporization in vivo. Ultimately, the experiment here helped develop the protocol used in the following in vivo drug-delivery experiment.

### 3.8. OFP-PDLC Mediated Fluorescein Delivery to Mock Tumors In Vivo

**Figure 10:**
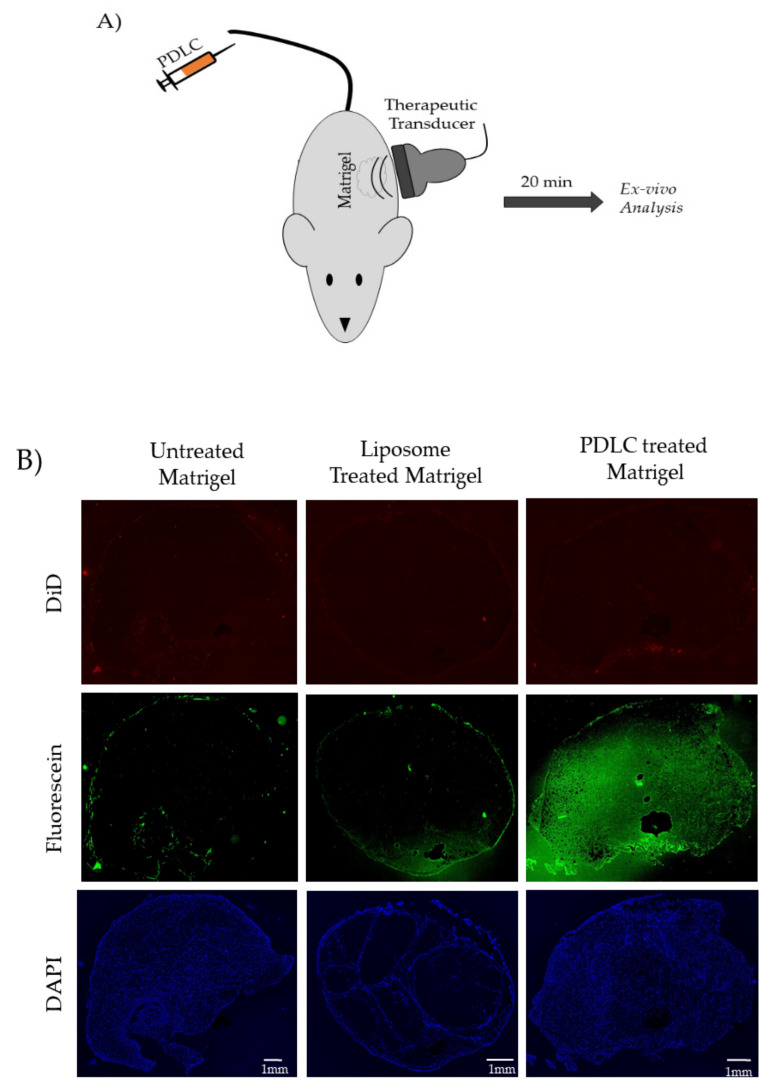
(**A**) Experimental setup for vaporizing PDLCs and trigger fluorescein uncaging (**B**) DAPI (cell nuclei), Fluorescein (model drug), and DiD (droplet shell) channels show the effect of uncaging and fluorescein delivery to Matrigel™ plugs. All scale bars show 1 mm.

Freshly prepared OFP-PDLCs were injected systemically in CD-1 mice bearing Matrigel™ “mock” tumors, as described in the methods section. Fluorescein was used in place of calcein in these studies. Otherwise, PDLC formation was identical. Matrigel™ plugs were excised and evaluated for fluorescein uptake. Whole slide microscopy of excised tissue sections was performed on untreated, fluorescein-liposome only treated (no sonication), and PDLC treated (with sonication), Matrigel™ plug “mock” tumors (Figure 10). A significant enhancement in the fluorescein intensity was observed in the Matrigel™ plugs treated with PDLCs and sonication compared to the untreated and liposome only treated samples. The in vivo studies did have some challenges. Systemic delivery of OFP-PDLCs needed to be performed at 4 °C to minimize spontaneous vaporization. The PDLCs would then rise to body temperature in circulation while sonication was applied. Interestingly, we were unable to visualize the vaporization in the Matrigel™ plugs when using a clinical scanner (data not shown). This observation was not consistent with our previous in vitro experiment. One possible reason is that the droplets were passing through the focal region in circulation, whereas all of the droplets were in a confined chamber for the in vitro studies. Despite the inability to observe the vaporization events on the clinical scanner, the fluorescein molecules were readily detectable in the excised Matrigel™ plug (Figure 10B Green channel). Matrigel™ treated with liposomes alone showed little fluorescein accumulation within the Matrigel™ plugs. No noticeable difference in DiD uptake (which labels the droplet shell) was observed between any of the treated groups, suggesting that fluorescein may be released systemically rather than through delivery of the whole liposome package. The mechanism of delivery is not established in these studies, however, which serve as proof-of-principal that PDLCs can effectively deliver packaged small molecule drugs using focused ultrasound in vivo.

## 4. Conclusions and Future Direction

In this study, we developed a strategy to conjugate nanoscale droplets to liposome nanoparticles, which are more amenable to drug loading. The goal of the study was to demonstrate efficient release of small molecules encapsulated in liposomes using droplet vaporization as a trigger for on-demand release. We used two low-boiling point droplet formulations, DFB and OFP, that are frequently used in vivo data due to their balance between shelf-stability and vaporization efficiency at body temperature. OFP droplets showed more efficient vaporization at 37 °C and higher total release of encapsulated calcein and are better candidates for focused ultrasound-mediated drug delivery in vivo. It should be noted, though, handling of the OFP-PDLCs (much like OFP droplets) requires extra care and cold conditions to minimize spontaneous vaporization and premature drug release before use. Methods of tuning the droplet vaporization threshold are needed for the wide-spread use of PDLCs. 

Our in vivo shows that OFP droplets can deliver packaged fluorescein molecules more efficiently than EPR-reliant circulating liposomes. However, it is unclear how much of the delivery may be due to systemic uncaging of the molecules vs. permeabilization of the tissue (artificially enhanced EPR effect). Further work in real tumor models will be needed to discriminate the dominant mechanism of uptake. Regardless of the mechanism, based on our in vitro drug release data, it is expected that a significant amount of an encapsulated drug would become bioavailable on-demand, which could potentially enhance the therapy of a broad class of drug molecules. 

In addition to drug delivery, we propose PDLCs can take advantage of the versatility of liposome nanocarriers to create multi-modal therapeutic and/or imaging contrast agents. Clustering liposomes encapsulated with MR or CT imaging contrast agents can potentially increase their clinical utility with minimal effort to create such constructs. Future experiments will focus on exploring novel applications for image-guided delivery of drugs using PDLC constructs.

## Data Availability

The data in this study are available on request from the corresponding author.

## Supplementary Materials

The following are available online at https://www.mdpi.com/article/10.3390/pharmaceutics13050609/s1, Video S1: Vaporization video and calcein release from DFB PDLCs inside of a capillary tube after a single pulse train (300 cycles,1.85 MPa, 1.1 MHz). (A) Brightfield microscopy. Droplets vaporize immediately after US pulse train. Formed bubbles rise to the top due to their buoyancy and coalesce to form lager bubbles. (B) Fluorescence microscopy. Immediate increase in fluorescence after US pulse train is indicative of rapid release of encapsulated calcein. Video S2: Visualizing focused ultrasound induced OFP droplet vaporization using a clinical scanner; Figure S1. Comparing the acoustic activation threshold of phase changeable droplets when conjugated to liposomes versus unconjugated phase changeable droplets.

## Abbreviations

Abbreviation	Full name
ADV	Acoustic Droplet Vaporization
DFB	Decafluorobutane
DLS	Dynamic Light Scattering
HCC	Hepatocellular Carcinoma
OFP	Octafluoropropane
PCA	Phase-shift Contrast Agents
PDLC	Phase shift Droplet-Liposome Cluster
PFC	Perfluorocarbon
PNP	Peak Negative Pressure
SPAAC	Single-step strain-Promoted [3 + 2] Azide-Alkyne Cycloaddition
UCA	Ultrasound Contrast Agents
US	Ultrasound

## References

[1] Mura S., Nicolas J., Couvreur P. (2013). Stimuli-Responsive Nanocarriers for Drug Delivery. Nat. Mater..

[2] Dong X. (2018). Current Strategies for Brain Drug Delivery. Theranostics.

[3] Danhier F., Feron O., Préat V. (2010). To Exploit the Tumor Microenvironment: Passive and Active Tumor Targeting of Nanocarriers for Anti-Cancer Drug Delivery. J. Control. Release.

[4] Matsumura Y., Maeda H. (1986). A New Concept for Macromolecular Therapeutics in Cancer Chemotherapy: Mechanism of Tumoritropic Accumulation of Proteins and the Antitumor Agent Smancs. Cancer Res..

[5] Iyer A.K., Khaled G., Fang J., Maeda H. (2006). Exploiting the Enhanced Permeability and Retention Effect for Tumor Targeting. Drug Discov. Today.

[6] Bobo D., Robinson K.J., Islam J., Thurecht K.J., Corrie S.R. (2016). Nanoparticle-Based Medicines: A Review of FDA-Approved Materials and Clinical Trials to Date. Pharm. Res..

[7] Wicki A., Witzigmann D., Balasubramanian V., Huwyler J. (2015). Nanomedicine in Cancer Therapy: Challenges, Opportunities, and Clinical Applications. J. Control. Release.

[8] Bazak R., Houri M., Achy S.E., Hussein W., Refaat T. (2014). Passive Targeting of Nanoparticles to Cancer: A Comprehensive Review of the Literature. Mol. Clin. Oncol..

[9] Park K. (2013). Facing the Truth about Nanotechnology in Drug Delivery. ACS Nano.

[10] Venditto V.J., Szoka F.C. (2013). Cancer Nanomedicines: So Many Papers and so Few Drugs!. Adv. Drug Deliv. Rev..

[11] Danhier F. (2016). To Exploit the Tumor Microenvironment: Since the EPR Effect Fails in the Clinic, What Is the Future of Nanomedicine?. J. Control. Release.

[12] Nichols J.W., Bae Y.H. (2014). EPR: Evidence and Fallacy. J. Control. Release.

[13] Maeda H. (2015). Toward a Full Understanding of the EPR Effect in Primary and Metastatic Tumors as Well as Issues Related to Its Heterogeneity. Adv. Drug Deliv. Rev..

[14] Ekdawi S.N., Jaffray D.A., Allen C. (2016). Nanomedicine and Tumor Heterogeneity: Concept and Complex Reality. Nano Today.

[15] Chauhan V.P., Jain R.K. (2013). Strategies for Advancing Cancer Nanomedicine. Nat. Mater..

[16] Rosenblum D., Joshi N., Tao W., Karp J.M., Peer D. (2018). Progress and Challenges towards Targeted Delivery of Cancer Therapeutics. Nat. Commun..

[17] Nakamura Y., Mochida A., Choyke P.L., Kobayashi H. (2016). Nanodrug Delivery: Is the Enhanced Permeability and Retention Effect Sufficient for Curing Cancer?. Bioconjug. Chem..

[18] Gasselhuber A., Dreher M.R., Rattay F., Wood B.J., Haemmerich D. (2012). Comparison of Conventional Chemotherapy, Stealth Liposomes and Temperature-Sensitive Liposomes in a Mathematical Model. PLoS ONE.

[19] Needham D., Anyarambhatla G., Kong G., Dewhirst M.W. (2000). A New Temperature-Sensitive Liposome for Use with Mild Hyperthermia: Characterization and Testing in a Human Tumor Xenograft Model. Cancer Res..

[20] Ta T., Porter T.M. (2013). Thermosensitive Liposomes for Localized Delivery and Triggered Release of Chemotherapy. J. Control. Release.

[21] Koning G.A., Eggermont A.M.M., Lindner L.H., ten Hagen T.L.M. (2010). Hyperthermia and Thermosensitive Liposomes for Improved Delivery of Chemotherapeutic Drugs to Solid Tumors. Pharm. Res..

[22] Landon C.D. (2011). Nanoscale Drug Delivery and Hyperthermia: The Materials Design and Preclinical and Clinical Testing of Low Temperature-Sensitive Liposomes Used in Combination with Mild Hyperthermia in the Treatment of Local Cancer. TONMJ.

[23] Tak W.Y., Lin S.-M., Wang Y., Zheng J., Vecchione A., Park S.Y., Chen M.H., Wong S., Xu R., Peng C.-Y. (2018). Phase III HEAT Study Adding Lyso-Thermosensitive Liposomal Doxorubicin to Radiofrequency Ablation in Patients with Unresectable Hepatocellular Carcinoma Lesions. Clin. Cancer Res..

[24] Lyon P.C., Griffiths L.F., Lee J., Chung D., Carlisle R., Wu F., Middleton M.R., Gleeson F.V., Coussios C.C. (2017). Clinical Trial Protocol for TARDOX: A Phase I Study to Investigate the Feasibility of Targeted Release of Lyso-Thermosensitive Liposomal Doxorubicin (ThermoDox®) Using Focused Ultrasound in Patients with Liver Tumours. J. Ther. Ultrasound.

[25] Lyon P.C., Gray M.D., Mannaris C., Folkes L.K., Stratford M., Campo L., Chung D.Y.F., Scott S., Anderson M., Goldin R. (2018). Safety and Feasibility of Ultrasound-Triggered Targeted Drug Delivery of Doxorubicin from Thermosensitive Liposomes in Liver Tumours (TARDOX): A Single-Centre, Open-Label, Phase 1 Trial. Lancet Oncol..

[26] Dou Y., Hynynen K., Allen C. (2017). To Heat or Not to Heat: Challenges with Clinical Translation of Thermosensitive Liposomes. J. Control. Release.

[27] Qin S., Caskey C.F., Ferrara K.W. (2009). Ultrasound Contrast Microbubbles in Imaging and Therapy: Physical Principles and Engineering. Phys. Med. Biol..

[28] Epstein-Barash H., Orbey G., Polat B.E., Ewoldt R.H., Feshitan J., Langer R., Borden M.A., Kohane D.S. (2010). A Microcomposite Hydrogel for Repeated On-Demand Ultrasound-Triggered Drug Delivery. Biomaterials.

[29] Paul S., Nahire R., Mallik S., Sarkar K. (2014). Encapsulated Microbubbles and Echogenic Liposomes for Contrast Ultrasound Imaging and Targeted Drug Delivery. Comput. Mech..

[30] Kheirolomoom A., Dayton P.A., Lum A.F.H., Little E., Paoli E.E., Zheng H., Ferrara K.W. (2007). Acoustically-Active Microbubbles Conjugated to Liposomes: Characterization of a Proposed Drug Delivery Vehicle. J. Control. Release.

[31] Martin K.H., Dayton P.A. (2013). Current Status and Prospects for Microbubbles in Ultrasound Theranostics: Current Status and Prospects for Microbubbles. WIREs Nanomed. Nanobiotechnol..

[32] Klibanov A.L., Shevchenko T.I., Raju B.I., Seip R., Chin C.T. (2010). Ultrasound-Triggered Release of Materials Entrapped in Microbubble–Liposome Constructs: A Tool for Targeted Drug Delivery. J. Control. Release.

[33] Ozdas M.S., Shah A.S., Johnson P.M., Patel N., Marks M., Yasar T.B., Stalder U., Bigler L., von der Behrens W., Sirsi S.R. (2020). Non-Invasive Molecularly-Specific Millimeter-Resolution Manipulation of Brain Circuits by Ultrasound-Mediated Aggregation and Uncaging of Drug Carriers. Nat. Commun..

[34] Grayburn P. (1997). Perflenapent Emulsion (Echogen®): A New Long-Acting Phase-Shift Agent for Contrast Echocardiography. Clin. Cardiol..

[35] Kripfgans O.D., Fowlkes J.B., Miller D.L., Eldevik O.P., Carson P.L. (2000). Acoustic Droplet Vaporization for Therapeutic and Diagnostic Applications. Ultrasound Med. Biol..

[36] Sheeran P.S., Dayton P.A. (2012). Phase-Change Contrast Agents for Imaging and Therapy. CPD.

[37] Rapoport N. (2012). Phase-Shift, Stimuli-Responsive Perfluorocarbon Nanodroplets for Drug Delivery to Cancer: Phase-Shift Perfluorocarbon Nanoemulsions. WIREs Nanomed. Nanobiotechnol..

[38] Burgess M.T., Porter T.M. (2019). Control of Acoustic Cavitation for Efficient Sonoporation with Phase-Shift Nanoemulsions. Ultrasound Med. Biol..

[39] Mountford P.A., Smith W.S., Borden M.A. (2015). Fluorocarbon Nanodrops as Acoustic Temperature Probes. Langmuir ACS J. Surf. Colloids.

[40] Mountford P.A., Thomas A.N., Borden M.A. (2015). Thermal Activation of Superheated Lipid-Coated Perfluorocarbon Drops. Langmuir ACS J. Surf. Colloids.

[41] Sheeran P.S., Luois S., Dayton P.A., Matsunaga T.O. (2011). Formulation and Acoustic Studies of a New Phase-Shift Agent for Diagnostic and Therapeutic Ultrasound. Langmuir ACS J. Surf. Colloids.

[42] Seda R., Li D.S., Fowlkes J.B., Bull J.L. (2015). Characterization of Bioeffects on Endothelial Cells under Acoustic Droplet Vaporization. Ultrasound Med. Biol..

[43] Kang S.-T., Lin Y.-C., Yeh C.-K. (2014). Mechanical Bioeffects of Acoustic Droplet Vaporization in Vessel-Mimicking Phantoms. Ultras. Sonochem..

[44] Fix S.M., Novell A., Yun Y., Dayton P.A., Arena C.B. (2017). An Evaluation of the Sonoporation Potential of Low-Boiling Point Phase-Change Ultrasound Contrast Agents in Vitro. J. Ther. Ultrasound.

[45] Wu S.-Y., Fix S.M., Arena C.B., Chen C.C., Zheng W., Olumolade O.O., Papadopoulou V., Novell A., Dayton P.A., Konofagou E.E. (2018). Focused Ultrasound-Facilitated Brain Drug Delivery Using Optimized Nanodroplets: Vaporization Efficiency Dictates Large Molecular Delivery. Phys. Med. Biol..

[46] Rapoport N., Gao Z., Kennedy A. (2007). Multifunctional Nanoparticles for Combining Ultrasonic Tumor Imaging and Targeted Chemotherapy. J. Natl. Cancer Inst..

[47] Airan R.D., Meyer R.A., Ellens N.P.K., Rhodes K.R., Farahani K., Pomper M.G., Kadam S.D., Green J.J. (2017). Noninvasive Targeted Transcranial Neuromodulation via Focused Ultrasound Gated Drug Release from Nanoemulsions. Nano Lett..

[48] Zhong Q., Yoon B., Aryal M., Wang J., Karthik A., Airan R. (2019). Polymeric Perfluorocarbon Nanoemulsions Are Ultrasound-Activated Wireless Drug Infusion Catheters. Biomaterials.

[49] Rapoport N.Y., Kennedy A.M., Shea J.E., Scaife C.L., Nam K.-H. (2009). Controlled and Targeted Tumor Chemotherapy by Ultrasound-Activated Nanoemulsions/Microbubbles. J. Control. Rel..

[50] Sheeran P.S., Luois S.H., Mullin L.B., Matsunaga T.O., Dayton P.A. (2012). Design of Ultrasonically-Activatable Nanoparticles Using Low Boiling Point Perfluorocarbons. Biomaterials.

[51] Sheeran P.S., Wong V.P., Luois S., McFarland R.J., Ross W.D., Feingold S., Matsunaga T.O., Dayton P.A. (2011). Decafluorobutane as a Phase-Change Contrast Agent for Low-Energy Extravascular Ultrasonic Imaging. Ultrasound Med. Biol..

[52] Chen C.C., Sheeran P.S., Wu S.-Y., Olumolade O.O., Dayton P.A., Konofagou E.E. (2013). Targeted Drug Delivery with Focused Ultrasound-Induced Blood-Brain Barrier Opening Using Acoustically-Activated Nanodroplets. J. Control. Rel..

[53] Chen C.C., Borden M.A. (2010). Ligand Conjugation to Bimodal Poly(Ethylene Glycol) Brush Layers on Microbubbles. Langmuir.

[54] Gaber M.H., Wu N.Z., Hong K., Huang S.K., Dewhirst M.W., Papahadjopoulos D. (1996). Thermosensitive Liposomes: Extravasation and Release of Contents in Tumor Microvascular Networks. Int. J. Radiat. Oncol. Biol. Phys..

[55] Maherani B., Arab-Tehrany E., Kheirolomoom A., Geny D., Linder M. (2013). Calcein Release Behavior from Liposomal Bilayer; Influence of Physicochemical/Mechanical/Structural Properties of Lipids. Biochimie.

[56] Hall R.L., Juan-Sing Z.D., Hoyt K., Sirsi S.R. (2019). Formulation and Characterization of Chemically Cross-Linked Microbubble Clusters. Langmuir.

[57] Lindsey B.D., Rojas J.D., Dayton P.A. (2015). On the Relationship Between Microbubble Fragmentation, Deflation and Broadband Superharmonic Signal Production. Ultrasound Med. Biol..

[58] Bellary A., Villarreal A., Eslami R., Undseth Q.J., Lec B., Defnet A.M., Bagrodia N., Kandel J.J., Borden M.A., Shaikh S. (2020). Perfusion-Guided Sonopermeation of Neuroblastoma: A Novel Strategy for Monitoring and Predicting Liposomal Doxorubicin Uptake in vivo. Theranostics.

[59] Slagle C.J., Thamm D.H., Randall E.K., Borden M.A. (2018). Click Conjugation of Cloaked Peptide Ligands to Microbubbles. Bioconjugate Chem..

[60] Correas J.M., Quay S.D. (1996). EchoGen Emulsion: A New Ultrasound Contrast Agent Based on Phase Shift Colloids. Clin. Radiol..

[61] Hassan P.A., Rana S., Verma G. (2015). Making Sense of Brownian Motion: Colloid Characterization by Dynamic Light Scattering. Langmuir.

[62] Chattaraj R., Mohan P., Besmer J.D., Goodwin A.P. (2015). Selective Vaporization of Superheated Nanodroplets for Rapid, Sensitive, Acoustic Biosensing. Adv. Healthcare Mater..

[63] Reznik N., Shpak O., Gelderblom E.C., Williams R., de Jong N., Versluis M., Burns P.N. (2013). The Efficiency and Stability of Bubble Formation by Acoustic Vaporization of Submicron Perfluorocarbon Droplets. Ultrasonics.

[64] De Gracia Lux C., Vezeridis A.M., Lux J., Armstrong A.M., Sirsi S.R., Hoyt K., Mattrey R.F. (2017). Novel Method for the Formation of Monodisperse Superheated Perfluorocarbon Nanodroplets as Activatable Ultrasound Contrast Agents. RSC Adv..

