# Supplementary material for: Improving Release of Liposome-Encapsulated Drugs with Focused Ultrasound and Vaporizable Droplet-Liposome Nanoclusters
Source: Pharmaceutics. 2021 Apr 22;13(5):609. doi: 10.3390/pharmaceutics13050609 (PMC8145150; doi:10.3390/pharmaceutics13050609)
Supplement: Supplementary file 1 [file pharmaceutics-13-00609-s001.zip › pharmaceutics-1138760 - supplementary for XML.pdf]

pharmaceutics

MDPI

Supplementary Materials: Improving Release of Liposome-Encapsulated Drugs with Focused Ultrasound and Vaporizable Droplet-liposome Nanoclusters

Arvin Honari, Darrah A. Merillat, Aditi Bellary, Mohammadaref Ghaderi and Shashank R. Sirsi

Publisher's Note: MDPI stays neutral with regard to jurisdictional claims in published maps and institutional affiliations.

Copyright: © 2020 by the authors. Submitted for possible open access publication under the terms and conditions of the Creative Commons Attribution (CC BY) license (http://creativecommons.org/licenses/by/4.0/).

Activation threshold measurement:

To assess the effect of liposomal conjugation on the vaporization threshold of droplets, standard grayscale ultrasound imaging was performed to measure the activation threshold of PDLCs and unconjugated droplets and liposomes. DFB PDLCs and DBCO blocked DFB droplets and liposomes were created as mentioned before. As shown in supplementary figure 1, a pipette bulb was immersed in a temperature controlled bath (45°C) and the PDLC solution was transferred to the pipette bulb. Acuson Sequoia 512 ultrasonography system (Siemens Medical Solutions, Erlangen, Germany), equipped with a linear 15L8 transducer operating at 8MHz was used to take B-mode videos from droplets while the mechanical index was manually increased every 10 seconds. The videos were post-processed using NIH ImageJ software to measure the overall signal intensity in the pipette bulb to find the activation threshold. This experiment was done in triplicate.

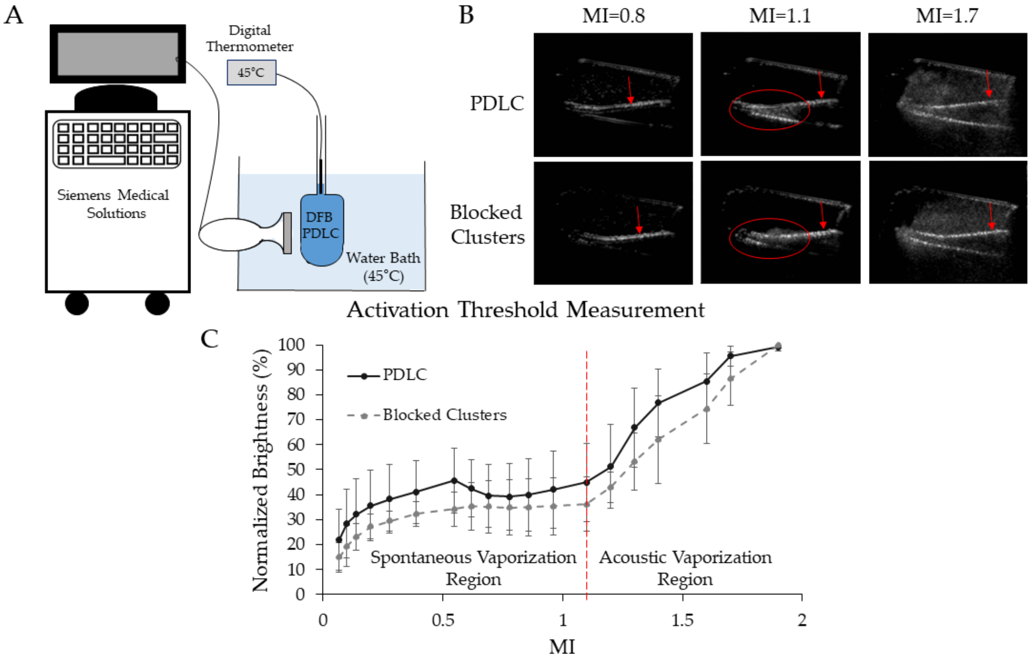

Figure S1. (**A**) Apparatus used for measuring activation threshold. (**B**) Representative images of B-mode videos before acoustic vaporization, right at the vaporization threshold and after vaporization threshold. The red ROIs show the onset of acoustic droplet vaporization. Red arrow in each image points at the temperature probe used for monitoring sample temperature (**C**) signal intensity in B-mode videos. By increasing the MI (ultrasound pressure) the signal intensity inside of the pipette bulb starts to increase at the threshold (MI = 1.1). This data suggests that both samples have the same activation threshold. At the MI highlighted as the threshold, a sudden increase of brightness is visible in both samples, indicating the initiation of acoustic droplet vaporization. Spontanious vaporizations can be observed before the initiation of ultrasound mediated vaporization at MIs lower than threshold.
